# Effects of diacetyl-liensinine on electrophysiology in rabbit ventricular myocytes

**DOI:** 10.1186/s40360-017-0137-6

**Published:** 2017-05-05

**Authors:** Feng Cao, Teng Wang, Wenmao Ding, Zhe Li, Shaobo Shi, Xiaozhan Wang

**Affiliations:** 1 Department of Cardiology, Renmin Hospital of Wuhan University, Wuhan, 430060 People’s Republic of China; 2 Cardiovascular Research Institute, Wuhan University, Wuhan, 430060 People’s Republic of China; 3 Hubei Key Laboratory of Cardiology, Wuhan, 430060 People’s Republic of China

**Keywords:** Diacetyl-liensinine, Ventricular myocyte, Electrophysiology, Patch clamp

## Abstract

**Background:**

Diacetyl-liensinine is a chemosynthetic derivative of liensinine, extracted from the seed embryo of *Nelumbo nucifera Gaertn*, in China. It has been found to have extensive anti- arrhythmic actions. The present study was designed to investigate the effects of diacetyl-liensinine on electro- physiology of myocytes.

**Methods:**

We exposed rabbit ventricular myocytes to diacetyl-liensinine using standard whole-cell patch-clamp technique and measured the action potential, L-type calcium current (*I*_Ca-L_), delayed rectifier potassium current (*I*_K_), transient outward potassium current (*I*_to_) and inward rectifier potassium current (*I*_K1_).

**Results:**

Our results showed that diacetyl-liensinine significantly prolonged action potential duration at 50 and 90% repolarization (APD50, APD90), at 10 and 30 μM, while shortened APD50 and APD90 at 100 μM. In addition, diacetyl-liensinine inhibited the *I*Ca-L, *I*K, *I*_to_ and *I*K_1_ in a concentration-dependent manner.

**Conclusions:**

The results suggest that diacetyl-liensinine might be a potential anti-arrhythmic agent.

## Background

Liensinine is a major effective bisbenzylisoquinoline alkaloid, which is extracted from the seed embryo of *Nelumbo nucifera Gaertn*, in China. Some studies showed that liensinine was effective in antagonizing arrhythmias induced by ouabain, aconitine, and calcium chloride [1, 2]. Liensinine could decrease maximum velocity of 0 phase depolarization and resting potential, prolong action potential duration, and inhibit sodium and calcium channel currents in a dose-dependent manner [3]. Liensinine has been reported to block hERG channel [4]. However, liensinine is unstable and easy to be oxidated. Diacetyl-liensinine (molecular formula: C_37_H_42_N_2_O_6_) is a chemosynthetic derivative of liensinine. It is more stable, and it has also extensive antiarrhythmic actions [5].

Arrhythmias are prevalent among humans, which may occur not only in the setting of underlying heart diseases, but also in normal hearts. Arrhythmias are widely varied in clinical presentations, but they have similar electrophysiological properties at cellular level, including triggered activity, automaticity, and reentry. Cardiac myocytes are highly specialized cells, which are responsible for conduction of electrical impulses as well as mechanical contraction. The disorders of impulse formation and impulse conduction of cardiac myocytes may lead to arrhythmia. The movement of ions through the transmembrane ion channels in cardiac myocytes generates action potentials, which differs in different parts of heart, distinguished byan excitatory and a contractile system. This differentiation of the action potential shows different electrical characteristics in different parts of heart, therefore, important physiological differences appeared between excitatory cells and muscular cells [6, 7].

The present study aimed to investigate the effects of diacetyl-liensinine on action potentials, ion channel currents, including L-type calcium current (*I*_Ca-L_), delayed rectifier potassium current (*I*_K_), transient outward potassium current (*I*_to_) and inward rectifier potassium current (*I*_K1_). It may be helpful for a deeply understanding of the ionic mechanisms underlying anti-arrhythmic actions of diacetyl-liensinine.

## Methods

### Solutions

The composition of Tyrode’s solution for perfusing the hearts was (mmol/L): NaCl 135, KCl 5.4, CaCl_2_ 1.8, MgCl_2_ 1.0, NaH_2_PO4 0.33, HEPES 10, Glucose 10, pH was adjusted with NaOH to 7.4. The calcium-free solution was the same as above except lacking CaCl_2_. The electrode solution for recording*I*_Ca-L_ contained (mmol/L): CsCl 120, CaCl_2_ 1, MgCl_2_ 5, Na_2_ATP 5.0, EGTA 11, HEPES 10, Glucose 11, pH was adjusted with CsOH to 7.4. Extracellular solution for recording potassium currents was the calcium-free Tyrode’s solution. The electrode solution for recording potassium currents contained (mmol/L): KCl 45, K-aspartate 85, Na-pyruvate 5, MgATP 5.0, EGTA 10, HEPES 10, Glucose 11, pH was adjusted with KOH to 7.4. For *I*_to_ and *I*_Ks_ determination, 500 μM BaCl_2_ and 100 μM CdCl_2_ were added to the bath solution to block *I*_K1_ and *I*_Ca,L_ [1].

### Drugs/reagents

Collagenase type-I, HEPES, Na_2_ATP, EGTA, BSA, MgATP and CsOH were obtained from Sigma (USA). Diacetyl-liensinine was provided by Pharmaceutical College of Wuhan University (China). Water was vehicle in which diacetyl-liensinine was dissolved as concentrated stock solutions. All drugs were diluted to their final concentrations in the bath solution immediately prior to the experiments.

### Cell isolation

Adult New Zealand White rabbits weighing 1.5 – 2.0 kg were used in the present study. Single ventricular myocytes were isolated by enzymatic dissociation method as reported previously [8, 9]. The rabbits were heparinized with 500U/kg heparin i.v., and anesthetized using 30 mg/kg pentobarbital. The heart was rapidly removed via thoracotomy and put into oxygenated calcium free Tyrode’s solution. The aorta was cannulated and the heart was perfused on Langendorff apparatus at 37 °C. Following perfusion with calcium-free Tyrode’s solution for about 3 min, the low calcium (100 μmol/L) Tyrode’s solution containing 0.33 mg/ml type-I collagenase was used for about 8 min. The ventricles were chopped, minced, and gently agitated to obtain myocytes. Cells were filtered through a 150 μm nylon mesh and calcium concentration was gradually increased. Then cells were stored in Tyrode’s solution containing 0.4% bovine serum albumin (BSA) at room temperature (23 °C) for at least 1 h before use.

### Whole-cell patch-clamp recordings

The whole-cell voltage clamp method was used for recording membrane currents [9]. A small aliquot of the solution containing the isolated cells was put in an open perfusion chamber (1.5 ml) mounted on the stage of an inverted microscope (Olympus, 2 × 70-122, Japan). Myocytes were allowed to adhere to the bottom of the pool for 5–10 min and were then superfused at 2-3 ml/min with the bath solution. Only rod-shaped cells with clear striation were used for experiments. The pool was perfused with extracelluar solution. Microelectriodes were pulled with a microeletrode puller (Narishige, PP-83, Japan) and had a resistance of 3-5 MΩ when filled with electrode internal solution. After giga-seal was formed and the membrane was ruptured, action potential was recorded in mode of current clamp and currents were recorded in a voltage-clamp mode using a patch-clamp amplifier (EPC-9, Germany). Capacitive-transients and series resistance were compensated. Experimental protocols, data acquisition and storage were accomplished with pClamp8.0 (HEKA, Germany) running on a personal computer. All experiments were conducted at 20–23 °C. All experiments were performed in accordance with the US National Institute of Health’s Guide for the Care and Use of Laboratory Animals and approved by the Animal Research Committee of Wuhan University [1].

### Data analysis

All data were presented as mean ± S.E.M. Paired Student’s *t*-test was used to evaluate the statistical significance of differences between two groups, while one-way analysis of variance was used for multiple groups. A difference at *P* < 0.05 was considered statistically significant.

## Results

### Effect of diacetyl-liensinine on action potential duration

**Fig. 1:**
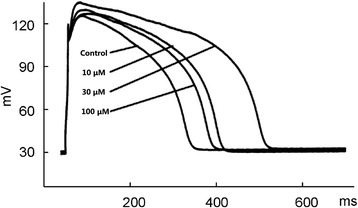
Effects of diacetyl-liensinine on action potentials in rabbit ventricular myocytes. Action potentials were elicited at a stimulation rate of 1 Hz and recorded in control and diacetyl-liensinine treatment (10, 30, 100 μM). Each experiment was performed with three replicates

Under the current clamp circumstance, the action potential was elicited by applying 900 pA, 15 ms duration stimuli at the frequency of 1 Hz [2]. As shown in Fig. 1, Diacetyl-liensinine at 10 and 30 μM prolonged the action potential duration at APD_50_ and APD_90_ (*n* = 9, *P* < 0.05), but shortened the action potential durationat 100 μM, which was also shown in Table 1.

**Table 1:** Effects of diacetyl-liensinine on APD_50_ and APD_90_

Concentrations	APD_50_ (ms)	APD_90_ (ms)
0 μM	245.48±122.32	310.51±69.82
10 μM	388.41±75.06^*^	417.37±45.79^*^
30 μM	479.08±81.11^**^	494.52±41.18^**^
100 μM	347.85±60.58^*^	358.01±53.16^*^

Data are expressed as mean±S.E.M. and compared by paired Student's *t*-test. (*n* = 9, *P <0.05, **P<0.01 vs. control) APD_50_: action potential duration at 50% repolarization; APD_90_: action potential duration at 50% repolarization

### Effect of diacetyl-liensinine on L-type calcium current

**Fig. 2:**
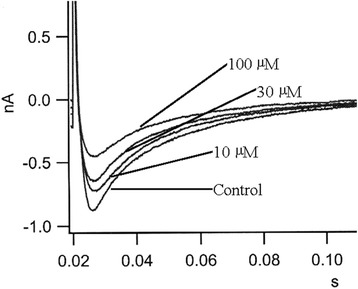
Inhibitory effects of diacetyl-liensinine with different concentration on the peak *I*_Ca,L_ in rabbit ventricular myocytes. The currents traces were evoked by 500 ms pulses between -30 and +60 mV from a holding potential of -40 mV in 10 mV increments. Each experiment was performed with three replicates

**Fig. 3:**
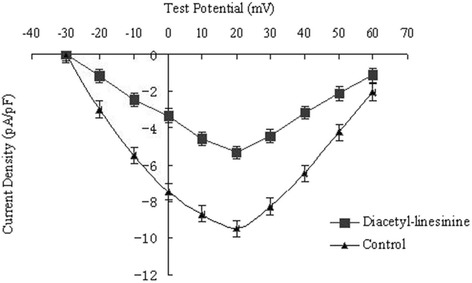
Effects of diacetyl-liensinine at 10 μM on *I-V* relationship for *I*_Ca,L_. Control was marked with closed triangle, diacetyl-liensinine at 10 μM with closed square. Five hundred milliseconds steps from a holding potantial of -40 mV were applied between -30 and +60 mV (increments of 10 mV) in seven cells of each group. Each experiment was performed with three replicates

*I*_Ca-L_ was elicited by 500 ms depolarization steps from the holding potential of -40 mV to test potentials of -30 mV to +60 mV in 10 mV increments. In order to avoid the “run-down” effects, *I*_Ca,L_ were measured between 5 and 15 min after rupturing the membrane patch in each cardiomyocyte from control and diacetyl-liensinine groups. When the testing potential was depolarized at +20 mV, *I*_Ca-L_ reached its peak. When cells were exposed to verapamil (1 μM), the elicited current was almost completely blocked [1]. As shown in Fig. 2, diacetyl-liensinine inhibited *I*_Ca-L_ in a concentration-dependent manner. Diacetyl-liensinine at 10, 30, and 100 μM reduced calcium current by 44.2%, 60.0% and 74.7% (from –9.51 ± 0.56 pA/pF to –5.31 ± 0.37 pA/pF, -3.78 ± 0.24 pA/pF and –2.37 ± 0.13 pA/pF), respectively (*n* =7, *P* < 0.05). The effect of diacetyl-liensinine on the current–voltage (*I–V*) relationship for the inward peak of *I*_Ca_was shown in Fig. 3. The data clearly showed that diacetyl-liensinine inhibited *I*_Ca-L_ at all the test potentials ranging from -30 to +60 mV, but did not affect the shape of its current-voltage curve.

### Effect of diacetyl-liensinine on the delayed rectifier potassium current

**Fig. 4:**
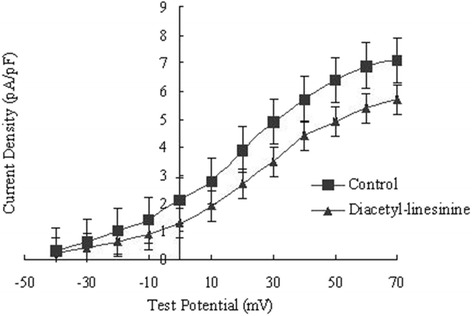
Effects of diacetyl-liensinine at 10 μM on the I-V relationship of IK. Control was marked with closed square, diacetyl-liensinine at 10 μM with closed triangle. Activation of IK was elicited by applying the voltage clamp steps at 0.1 Hz from a holding potential of -40 mV to depolarizing potentials ranging from -40 to +70 mV. Each experiment was performed with three replicates

The depolarization pulse of 5 s duration was used at various potentials from -40 mV to +70 mV with step of 10 mV to evoke *I*_K._ Diacetyl-liensinine (10-100 μM) caused a concentration- dependent inhibition of *I*_K_. The density of the peak current was decreased significantly from 7.13 ± 0.51 pA/pF in control to 5.69 ± 0.33 pA/pF at 10 μM, 3.43 ± 0.21 pA/pF at 30 μM and 2.54 ± 0.17 pA/pF at 100 μM of diacetyl-liensinine which reduced *I*_K_ by 19.7%, 52.1% and 64.8%at 10, 30, and 100 μM, respectively (n = 7, *P* < 0.05). The effect of diacetyl-liensinine on the current–voltage (*I–V*) relationship for *I*_K_was shown in Fig. 4. The data clearly showed that diacetyl-liensinine inhibited *I*_K_ at all the test potentials, but did not affect the shape of its current-voltage curve.

### Effect of diacetyl-liensinine on transient outward potassium current

Three cumulative concentrations of diacetyl-liensinine, 10, 30 and 100 μM, were tested on *I*_to_, with each concentration applied for 5 min. *I*_to_ was elicited by a series of 300 ms step depolarizing pulses applied at 0.1 Hz from a holding potential of -60 mV to test potentials between -50 mV to +60 mV during the infusion of 100 μM CdCl_2_ in the bath solution to inhibit *I*_Ca,L_ [1]. The density of the peak current was significantlydecreased from 9.80 ± 0.87 pA/pF (*n* = 7) in control to 6.51 ± 0.66 pA/pF (*P* < 0.05) at 10 μM, 4.87 ± 0.52 pA/pF (*P* < 0.05) at 30 μM and 3.65 ± 0.41 pA/pF (*P* < 0.01) at 100 μM of diacetyl-liensinine.

### Effect of diacetyl-liensinine on inward rectifier potassium current

*I*_K1_ was elicited by a number of step pulses (500 ms) from the holding potential of -40 mV to test potentials from -140 mV to +40 mV with 10 mV increments. The density of the peak inward current was significantlydecreased from 7.61 ± 0.66 pA/pF in control to 6.36 ± 0.52 pA/pF at 10 μM, 4.92 ± 0.31 pA/pF at 30 μM and 3.67 ± 0.26 pA/pF at 100 μM of diacetyl-liensinine (n = 7, *P* < 0.05).

## Discussion

The present study demonstrates that diacetyl-liensinine as a multi-channel blocker has direct electrophysiological effects on rabbit ventricular myocytes. A balanced inhibition of potassium and calcium channels resulted in a moderate prolongation of action potential duration [10, 11]. *I*_K_ is a major outward current in determining the action potential plateau in the myocardium [12, 13]. The prolongation of APD by diacetyl-liensinine at low concentrations (10, 30 μM) appears to be primarily due to an inhibition of *I*_K_. The blocking effects of diacetyl-liensinine on potassium channels may lead to a further prolongation of APD at high concentrations. However, a concomitant block of *I*_Ca_ might limit the increase in APD at high concentrations (100 μM).

It has been repored that there are two types (L and T) calcium channels in cardiac myocytes. Under the condition of individual cell depolarization from holding potential of –40 mV, the L-type calcium channel was activated, while T-type calcium channel and sodium channel were inactivated [14–16]. Furthermore, verapamil, a typical L-type calcium channel antagonist, could inhibit the recorded current. Therefore, L-type calcium current was recorded as the inward current under these conditions. In this study, diacetyl-liensinine obviously suppressed the *I*_Ca-L._ What’s more, *I-V* relationship of *I*_Ca-L_ indicated that the peak calcium current was decreased by diacetyl-liensinine at all depolarizing potentials. However, there were no change in the activated potential, peak amplitude potential, as well as the reversal potential of *I*_Ca-L_. This indicated that the blocking effect of diacetyl-liensinine on calcium channel was voltage-independent.

Our results showed that diacetyl-liensinine at 10 and 30 μM significantly blocked *I*_Ca-L_, *I*_K_, *I*_to_ and *I*_K1_. The “run-down” phenomenon has long been noticed in recording L-type calcium current [16]. Generally speaking, it decreases relatively slowly within 20-60 min and then abruptly reduces to zero. To supply the pipette solution with ATP can double the survival time. In our experiment, drugs at low concentrations were added within 10 min and high concentration within 20 min. So in order to distinguish the decreased effect caused by drugs from the “run-down” phenomenon, we compared the drug groups with controls at 10 and 20 min, respectively. Only when there was significant difference, the decreased effects of the drugs were confirmed. As we all know, calcium overload is the main underlying mechanism for myocardial ischemia and reperfusiun injury [17]. Yu et al. suggested that I_Ca_ blockade couldconfer cardioprotective effect on the non ischemic heart [18]. What’s more, calcium antagonists had a positive role on ischemic and reperfused myocardium. Because diacetyl-liensinine could inhibit the calcium channel in myocytes, the intracellular calcium concentration was reduced due to the decrease of calcium influx. Therefore, diacetyl-liensinine maintained the integrity of myofibrillar membrane and minimized ATP depletion through attenuating calcium overload.

Present class III antiarrhythmic compounds can prolong APD and ERP of cardiomyocytes, which are mainly due to the depression of *I*_K_. However, these drugs are thought to be associated with arrhythmogenic phenomena, such as long QT syndrome and torsade de pointes [19]. Therefore, in order to avoid these adverse effects, we should make efforts to study and develop anti-arrhythmic drugs that can block multi-ionic channels.

In the present study, we demonstrated the inhibitory effects of diacetyl-liensinine on *I*_Ca-L_, *I*_to_, *I*_K_ and *I*_K1_ in ventricular myocytes of rabbit in a concentration-dependent manner. *I*_K1_ and *I*_K_ channels of myocardium are very important in maintaining normal rest potential and APD in electrophysiology of heart [20, 21]. Some studies reported that the blockage of potassium channels could protect myocytes against arrhythmia. The inhibitory effects of diacetyl-liensinine on *I*_K1_ ang *I*_K_ may be another ionic basis of antagonizing experimental arrhythmia, besides *I*_Ca-L_ channel blockage.

There are still several limitations in our research. Firstly, the existence of run-down of the potassium and calcium currents may cause an overestimation of the blocking effects of diacetyl-liensinine on these currents. To minimize the influence of rundown, we selected the cells with good seals and stable recordings. Secondly, since *I*_K_ is very sensitive to isolation procedures, changes in isolation technique could lead to differences in *I*_K_. Therefore, in order to minimize possible effects of time-dependent changes in enzymes, isolation procedure, and so on, animals from each group were researched concurrently in an alternative fashion [22].

## Conclusions

In conclusion, diacetyl-liensinine resulted in a modest prolongation of APD through inhibiting both potassium and calcium currents, which may lead to a higher efficiency in the treatment of arrhythmias and lower risk of proarrhythmic agents, supplying an underlying pharmacological rationale for drugs used in the treatment of ventricular arrhythmias. It is a potential novel drug to treat arrhythmia; however, other pharmacodynamics, pharmacokinetics, and toxicology of diacetyl-liensinine need to be further explored.

## Abbreviations

APD: Action potential duration
BSA: Bovine serum albumin
*I*_Ca-L_: L-type calcium current
*I*_K_: Delayed rectifier potassium current
*I*_K1_: Inward rectifier potassium current
*I*_to_: Transient outward potassium current
*I–V*: Current–voltage
